# Do invasive cane toads affect the parasite burdens of native Australian frogs?^☆^

**DOI:** 10.1016/j.ijppaw.2013.04.002

**Published:** 2013-04-22

**Authors:** Damian C. Lettoof, Matthew J. Greenlees, Michelle Stockwell, Richard Shine

**Affiliations:** a School of Biological Sciences A08, University of Sydney, NSW 2006, Australia; b School of Biological Sciences, University of Newcastle, Callaghan, NSW 2308, Australia

**Keywords:** Host–parasite interaction, Host-switch, Novel host, Biological invasion, Anura

## Abstract

One of the most devastating impacts of an invasive species is the introduction of novel parasites or diseases to native fauna. Invasive cane toads (*Rhinella marina*) in Australia contain several types of parasites, raising concern that the toads may increase rates of parasitism in local anuran species. We sampled cane toads and sympatric native frogs (*Limnodynastes peronii*, *Litoria latopalmata*, and *Litoria nasuta*) at the southern invasion front of cane toads in north-eastern New South Wales (NSW). We dissected and swabbed these anurans to score the presence and abundance of nematodes (*Rhabdias* lungworms, and gastric encysting nematodes), myxozoans, and chytrid fungus. To determine if cane toad invasion influences rates of parasitism in native frogs, we compared the prevalence and intensity of parasites in frogs from areas with toads, to frogs from areas without toads. Contrary to the situation on the (rapidly-expanding) tropical invasion front, cane toads on the slowly-expanding southern front were heavily infected with rhabditoid lungworms. Toads also contained gastric-encysting nematodes, and one toad was infected by chytrid fungus, but we did not find myxozoans in any toads. All parasite groups were recorded in native frogs, but were less common in areas invaded by toads than in nearby yet to be invaded areas. Contrary to our predictions, toad invasion was associated with a reduced parasite burden in native frogs. Thus, cane toads do not appear to transfer novel parasites to native frog populations, or act as a reservoir for native parasites to ‘spill-back’ into native frogs. Instead, cane toads may reduce frog-parasite numbers by taking up native parasites that are then killed by the toad’s immune defences.

## Highlights

• We examine cane toads from the southern invasion front for parasites and pathogens.
• We compare the parasitism of native Australian frogs sympatric with cane toads.
• Invading cane toads do alter the parasites or pathogens of native Australian frogs.

## Graphical abstract

**Figure:**
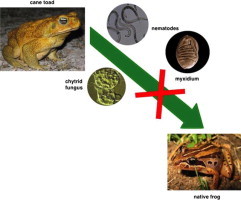

## 1 Introduction

Anthropogenic activities have transported many species into new habitats. When an exotic species establishes successfully, its effects on the newly-invaded ecosystem can be catastrophic (Zavaleta et al., 2001; Clavero and García-Berthou, 2005; Hartigan et al., 2011; Pizzatto and Shine, 2011). Australia has experienced negative impacts from many invasive species. The Australian fauna has suffered from introduced predators (e.g. foxes: Dickman, 1996), habitat modifiers and competitors (e.g. European rabbits: Eldridge and Myers, 2001; Moseby et al., 2009), and lethally toxic novel prey items (e.g. cane toads: Shine, 2010). Most research on the impacts of invasive species has focused on the direct ecological damage that they cause, via processes such as predation, competition, and lethal toxic ingestion (references above). Indirect impacts – such as the transmission of diseases and parasites – may be important also. For example, parasitic larvae of the fly *Philornis downsi*, native to mainland South America, were introduced to the Galápagos Islands and now infest most nests of endangered Darwin’s finches, reducing hatchling survival (Fessl et al., 2001, 2006; Fessl and Tebbich, 2002).

The interspecific transfer of pathogens and parasites from invasive species to native fauna is likely to be rare, because most pathogens and parasites are host-specific (Poulin, 2007; Pizzatto and Shine, 2011). However, parasites have shorter generation times than their hosts, heightening their advantage in the evolutionary arms race, and often allowing a quick counter-adaption to new host resistance strategies (Kaltz and Shykoff, 1998). The co-introduction of novel parasites with an invasive species can result in several outcomes: (a) the parasite remains restricted to the introduced species, and does not spread to local taxa; (b) the exotic host spreads the novel parasite to natives (Torchin et al., 2003; Prenter et al., 2004; Dubey and Shine, 2008), for example cattle and sheep liver fluke introduced to Australia with livestock now utilise marsupial hosts (Spratt and Presidente, 1981); (c) the invader serves as an additional host for an already-present parasite of the local fauna, increasing rates and intensities of parasitism in native taxa via ‘spill-back’ (Daszak et al., 2000; Pizzatto and Shine, 2011); and/or (d) the invader actually reduces parasite burdens of local fauna, by acting as a sink for native parasite populations (Kelly et al., 2009).

Introduced populations are usually produced from a small number of founding individuals, inevitably comprising only a small fraction of the diversity within native populations. Hence, the founding population is unlikely to contain all of the parasites present within the host’s native range (Barton, 1997; Torchin et al., 2003; Phillips et al., 2010). Because high parasite loads are likely to reduce the effectiveness of spread, successful invasive populations often harbour few parasites (Prenter et al., 2004). The translocation of species often acts as an inadvertent quarantine period (Barton, 1997; Dunn, 2009): heavily infected individuals are likely to perish before they can establish in a new environment, and lightly infected individuals may shed their infection before reaching their new home. Also, many parasites require multiple hosts to complete their complex life cycles (Fürst et al., 2012). If suitable intermediate hosts are inaccessible (which may often be the case during translocation), then such parasites may be extirpated from the founding population (Ginetsinkaya, 1988; Barton, 1997; Torchin et al., 2003). Even if invasive populations retain their native-range parasites, the process of biological invasion will tend to leave their parasites behind because low host population density at the front reduces parasite transmission rates (Arneberg et al., 1998; Phillips et al., 2010; Kelehear et al., 2012). This tendency is exacerbated if parasites reduce host mobility: the invasion front comes to be dominated by faster-moving (and hence, parasite-free) hosts (Phillips et al., 2010; Kelehear et al., 2012).

The transfer of parasites may involve transmission from the native fauna to an invader, as well as vice versa. Because invaders have not co-evolved with parasites from the new (invaded) range, they may be more susceptible to those parasites. This process might reduce invader viability, and hence slow the advance of the invasion front (Dunn, 2009). Although many of the parasites adapted to native hosts are unlikely to pose significant threats to a novel (invading) host (Moret et al., 2007; Dunn, 2009), invading species are more likely to acquire native parasites than to introduce exotics (Barton, 1997; Torchin et al., 2003; Dubey and Shine, 2008; Kelehear and Jones, 2010). Two outcomes can occur after an introduced population acquires native parasites. Firstly, invasive populations that are frequently exposed to novel parasites may suffer depressed fitness. Secondly, native parasites may adapt to infect the introduced hosts, a process that can result in a previously uncommon parasite increasing in abundance within natives, as well as introduced hosts because of its newly-acquired reservoir host population (Delvinquier and Freeland, 1988; Barton, 1997).

The invasion of cane toads (*Rhinella marina*) through Australia provides a robust model system within which to explore the impacts of an alien species not only in terms of direct effects (Shine, 2010), but also in terms of pathogen and parasite transmission (Pizzatto and Shine, 2011; Kelehear et al., 2012). In tropical Australia, the cane toad invasion has moved with increasing speed, and currently averages around 50–60 km per year (Phillips et al., 2006). The resulting low transmission probability for parasites in frontal populations has resulted in invasion-front populations of toads lacking the native-range lungworm *Rhabdias pseudosphaerocephala* (Phillips et al., 2010; Kelehear et al., 2012). Surveys of lungworm faunas in native frogs and invasive toads in tropical northern Australia have shown that the toads’ lungworm has not transferred to infect frogs, nor have the frog lungworms (*Rhabdias* cf. *hylae*: Pizzatto and Shine, 2011) transferred to infect toads. In the current project, we examined parasite transmission at the other geographic extreme of the cane toads’ Australian invasion, in New South Wales (i.e. at the current southern front of toad expansion). There are two reasons why we might expect host-parasite interactions at this front to differ from those in tropical Australia. First, the southern front is expanding only slowly (<20 km per year: Urban et al., 2008) and thus, we would not expect the invasion-front toads to have left their parasites behind (unlike the situation with the fast-expanding tropical front). Second, the assemblage of native species, and the local abiotic (thermal, hydric) conditions in this region are very different from those in tropical Australia, so that local frogs might take up the “toad-specific” lungworm species (as does at least one tropical tree-frog taxon, at least in laboratory trials: Pizzatto et al., 2010) either because of species differences in vulnerability, or because cooler moister conditions allow longer survival of free-living life-history stages of the parasite life cycle (Kelehear et al., 2012).

The first step in understanding host–parasite interactions at the toads’ southern front is to survey the occurrence of parasites in both toads and frogs. In this study, we focused on several common parasites of cane toads and sympatric native frog species from the north coast of New South Wales (NSW). First, we determined the prevalence and intensity of parasites in cane toads, to test the prediction that toads would exhibit heavy parasite loads even at the invasion front (unlike the situation in tropical Australia). Second, we compared the prevalence and intensity of parasites in native frogs from areas where toads had recently invaded, to those of frogs in adjacent areas yet to be invaded by toads. If toads affect parasitism rates of native frogs (either by direct transmission or spillback), we expect to see higher rates of parasitism in frogs from areas that also contain toads, than from areas where toads have yet to arrive. Overall, our study was designed to clarify the consequences of cane toad invasion for host-parasite biology (of both toads and native frogs) in southern Australia.

## 2 Material and methods

### 2.1 Study area and species

#### 2.1.1 Anurans

Frogs and toads were obtained from roads adjacent to waterbodies (some of which contained cane toads, and some of which did not) between the towns Kingscliff and Broom’s Head, along the northern coast of NSW (Table 1). This region has a predominantly subtropical climate; mean minimum temperatures range from 9.7–21.0 °C in July/August to 26.7–31.3 °C in January, and mean monthly rainfall from 28.8 mm during winter to 233.1 mm in summer (Yamba, Casino Airport and Bray Park weather stations: Bureau of Meteorology, 2012). This area lies near the southernmost limit of the cane toad invasion front (Phillips et al., 2006), with toads distributed in a patchy fashion across the landscape.

In total, we obtained 44 road-killed toads and 100 road-killed native frogs (see Table 2 for list of these taxa, and a description of their habits and body sizes). Due to the ‘patchy’ distribution of toads in this area, toad-free sites were interspersed between sites where toads were present, so any patterns in parasite occurrence cannot be due to latitudinal or longitudinal confounding. Sites were considered toad-free if they were a minimum of 2 km from where any toads had been recorded (either during searches by DCL, or during extensive surveys over the past two years by MJG). Although we attempted to collect 10 specimens of each frog species from both toad-present and toad-absent sites, we were unable to do so: some species were not encountered often enough. Thus, our statistical analyses are based on the two frog species (*Limnodynastes peronii* and *Litoria latopalmata*) for which we were able to obtain robust sample sizes (Table 2).

#### 2.1.2 Nematodes

Parasitic nematodes are abundant and diverse (Procter, 1990; Roberts and Janovy, 2009; Blaxter et al., 2012). In anurans such as toads, a range of nematode species encyst as larvae on the gastric tissues (Kelehear and Jones, 2010) or live in the lungs as adults (Dubey and Shine, 2008; Pizzatto and Shine, 2011). Nematode infection can reduce the host’s fitness either due to damage during larval penetration, or as a result of the presence of the adult nematode within the host’s body (Baker, 1979; Pizzatto et al., 2010).

Rhabditoid lungworms are difficult to identify using morphological criteria, but DNA sequence data show that only a single species (*R. pseudosphaerocephala*) occurs in cane toads in Australia, whereas native frogs are infected by at least three species of the native lungworm, all currently lumped under the name *R.* cf. *hylae* (Dubey and Shine, 2008; Pizzatto et al., 2010). Although *R. pseudosphaerocephala* can infect native Australian frogs under laboratory conditions, it has not been detected in wild populations of frogs (Pizzatto et al., 2012). Thus, the lungworms detected in cane toads are likely to be *R. pseudosphaerocephala*, and the lungworms detected in native frogs are likely to be *R*. cf. *hylae*.

#### 2.1.3 Chytrid fungus

*Batrachochytrium dendrobatidis* is a waterborne fungus capable of infecting amphibians, and has caused a global epidemic disease referred to as ‘Chytridiomycosis’ (Longcore et al., 1999; McCallum, 2005; Kriger and Hero, 2006; Norris, 2007; Murray et al., 2009). *B. dendrobatidis* has two life stages; the motile infective zoospore and the stationary reproductive zoosporangium (Lam et al., 2011). Zoosporangia develop in the cutaneous keratin of amphibians and release zoospores in water (Piotrowski et al., 2004). A single zoospore can initiate an infection on the skin of a susceptible host (Lam et al., 2011). If an anuran infected with *B. dendrobatidis* survives, the host may lose the infection, or retain it and thereafter serve as a carrier of the fungus (Piotrowski et al., 2004).

#### 2.1.4 Myxozoans

Myxosporean parasites infect fish, amphibians and reptiles (Hill et al., 1997; Eiras, 2005). Ninety percent of 2200 described myxosporean species infect fish, yet the life cycles of only 33 species have been identified, none of them from anurans (Eiras, 2005; Hartigan et al., 2010). Nonetheless, because the described life-cycles all include an invertebrate host, this is likely to be true for the myxosporean parasites of amphibians and reptiles also (Eiras, 2005; Hartigan et al., 2012a). *Myxidium immersum* Lutz, 1889 (syn. *Cystodiscus immersus*, Lutz 1889) occurs in the gall bladders of cane toads (*R. marina*) and Australian frogs belonging to at least six families (Delvinquier, 1986; Eiras, 2005; Jirku et al., 2006).

### 2.2 Detection of parasites

Each road-killed anuran was placed into a separate clean 17 × 18 cm plastic, zip-lock bag. Prior to dissection, each specimen was measured (snout-urostyle length, SUL), weighed (g), and the sex determined (see Table 2 for data on sex ratios of samples). To ensure consistency in parasite detection the same researcher (DMCL) collected all data. In some cases, road-killed specimens were damaged in ways that prevented us from determining the sex (*n* = 3), or examining the lungs (*n* = 5), stomach (*n* = 3), or gall bladder (*n* = 21).

#### 2.2.1 *Rhabdias*

To detect *Rhabdias* lungworms, we removed and inspected the right lung of each anuran. Within an infected host, the numbers of *Rhabdias* are similar in the left and right lung (C. Kelehear, pers. comm.).

#### 2.2.2 Other nematodes

Parasitic nematodes have been reported within many organs of anurans (Zug and Zug, 1979; Yoder and Coggins, 2007; Kelehear and Jones, 2010; Fürst et al., 2012). We focussed upon gastric encysting nematode larvae, but included four un-encysted third-stage larvae (the infective stage of a parasitic nematode’s lifecycle) that we detected within the body cavities of two *L. peronii*.

#### 2.2.3 Chytrid fungus

Anurans were swabbed for *B. dendrobatidis* infection by firmly running a sterile cotton swab 10 times across the animal’s ventral surface, sides, groin and underside of foot (Kriger et al., 2006). Each specimen was handled with unused, unpowdered latex gloves to prevent cross-contamination of spores. Swabs were then returned to their original container (a plastic tube), and stored within a refrigerator for up to two months until analysis. Extraction and quantification of *B. dendrobatidis* on epidermal swabs was performed following standard procedures (Boyle et al., 2004), using a Rotor Gene 6000 real time DNA amplification system (Corbett Life Science, Mortlake, NSW). Each swab was analysed in triplicate alongside standards of known concentrations and negative controls to test for false positives. Where amplification did not occur in any of the replicates, the sample was considered negative for the presence of *B. dendrobatidis*, provided the PCR reaction was not inhibited. To detect inhibition (false negatives), internal positive controls were included in one replicate of each sample. Where inhibition was detected, a 1/100 dilution of the originally extracted DNA was prepared to dilute inhibitory agents and the reaction repeated. Where amplification occurred in any of the replicates, the mean number of genomic equivalents detected at a standardised cycle threshold was calculated, providing a relative measure of individual infection load.

#### 2.2.4 Myxozoans

To detect myxospores, the anuran gall bladders were removed and stored in 70% ethanol until examination. For processing, gall bladders were sliced open and placed on a wet mount and examined using a stereomicroscope with 20× and 40× objectives (SM1, Industrial and Scientific Supply Co., Concord West, NSW). Anurans were tested for presence or absence of myxospores; individual myxospores were not counted. Recently, two new myxosporean species (*Cystodiscus axonis* and *Cystodiscus australis*) have been discovered in the gall bladders of anurans on the east coast of Australia; they are morphologically indistinguishable from some other myxozoan species (Hartigan et al., 2012a). Because more precise identification of the myxosporean species requires genetic analysis, we recorded any myxospores as *Cystodiscus* sp.

### 2.3 Data analysis

Parasite prevalence for each anuran species was calculated as the number of individuals hosting parasites, divided by the number of individuals sampled (Margolis et al., 1982). Intensity of parasitism was calculated as the total number of adult worms in the lungs of an infected host (i.e. uninfected hosts were excluded). We also calculated a measure of overall parasite burden (mean number of parasites per host, including uninfected as well as infected hosts). We first performed contingency analyses to test for interspecific differences in the prevalence of each parasite or pathogen between toads and both species of native frog, irrespective of sympatry with toads. For the same groups, we used one-way ANOVAs to test for interspecific differences in intensity. Tukey’s HSD post-hoc tests were used to determine which groups were different.

To determine whether parasite prevalence in native frogs depended on whether or not the frogs were sympatric with cane toads, we performed logistic regression with independent variables of location (toads presence vs. absence) and frog phenotype (frog species, body mass), with presence or absence of the parasite as the dependent variable. Infection intensities were compared among species using one-factor ANOVA. In addition, to compare the mean intensity of parasites in native frogs between “toad-present” sites and “toad-absent” sites, we conducted ANOVAs with the same independent variables as above, plus the site was included as a random factor; the dependent variable was the number of parasites per host (excluding uninfected hosts, for our measure of intensity; but including all hosts, for our measure of overall parasite burden). All statistical tests were conducted using JMP Pro (version 9; SAS Institute, Cary, NC).

## 3 Results

Prevalence and intensity of parasites were higher in cane toads (*R. marina*) than in native frogs (Table 3). Contrary to our a priori prediction (that the invasion of toads might increase parasite burdens in native frogs), we found the opposite pattern. That is, native frogs (*L. peronii* and *L. latopalmata*) that were sympatric with cane toads had fewer, not more, parasites than did conspecific frogs that we collected from nearby sites where toads were absent. This comparison is based upon only two anuran species, because we were unable to obtain enough *Litoria nasuta* from “toad-present” sites.

### 3.1 *Rhabdias*

**Fig. 1:**
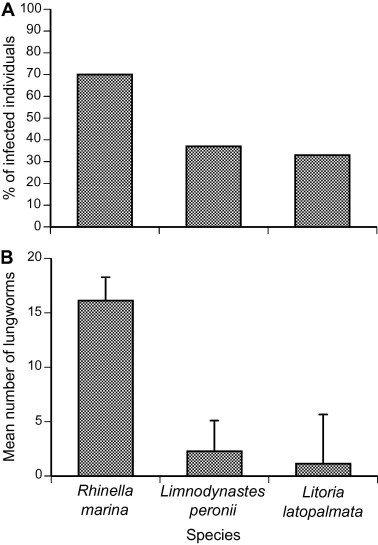
(A) Prevalence (% of anurans infected) and (B) intensity (mean number of worms per infected host) of parasitic lungworms in cane toads and native anuran from northern NSW. Bars represent standard errors.

**Fig. 2:**
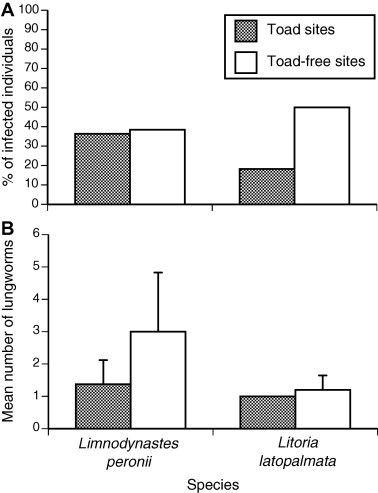
(A) Prevalence (% of anurans infected) and (B) intensity (mean number of worms per infected host) of parasitic lungworms in anurans from cane toad-present, and cane toad-absent areas in northern NSW. Bars represent standard errors.

Overall, the prevalence of adult *Rhabdias* lungworms differed significantly among anuran species (combining sites; *χ*^2^_2_ = 12.99, *p* < 0.005; Fig. 1). Cane toads were infected at higher intensities than were either *L. peronii* or *L. latopalmata* (one-factor ANOVA, *F*_2,53_ = 9.77, *p* < 0.001); the two native species were infected at similar intensities (Tukey’s HSD, *p* > 0.05). The mean intensity of lungworm infection was generally low in both native frogs (<5) and in cane toads (<20), although some cane toads contained more than 50 lungworms (Table 3). Both *L. peronii* and *L. latopalmata* exhibited a higher prevalence of lungworms at “cane toad-absent” sites than at “cane toad-present” sites (Fig. 2A). ANOVA on these data showed that whether or not a frog contained lungworms was affected by its body size (larger frogs were more likely to be infected; mass effect *χ*^2^_1_ = 6.42, *p* < 0.02) but not its species (*χ*^2^_1_ = 2.20, *p* = 0.14). Frogs from toad-invaded areas had a lower prevalence of lungworm infection (*χ*^2^_1_ = 4.32, *p* < 0.04). Lungworm intensity showed the same pattern as prevalence; that is, frogs from “cane toad-absent sites” contained more lungworms per infected host (Fig. 2B), as well as having a higher proportion of potential hosts infected. Mean intensity of infection per infected host did not differ significantly with respect to toad presence (*F*_1,1.19_ = 4.84, *p* = 0.24), frog species (*F*_1,8.48_ = 3.084, *p* = 0.12) or frog mass (*F*_1,9.50_ = 0.12, *p* = 0.74). The overall parasite burden due to *Rhabdias* infection was thus higher in frogs collected from “toad-present” versus “toad-absent” sites (ANOVA, effect of body size *F*_1,49.22_ = 6.42, *p* < 0.02; effect of species *F*_1,11.35_ = 0.14, *p* = 0.71; effect of toad presence *F*_1,8.22_ = 7.87, *p* < 0.025).

### 3.2 Other larval nematodes

**Fig. 3:**
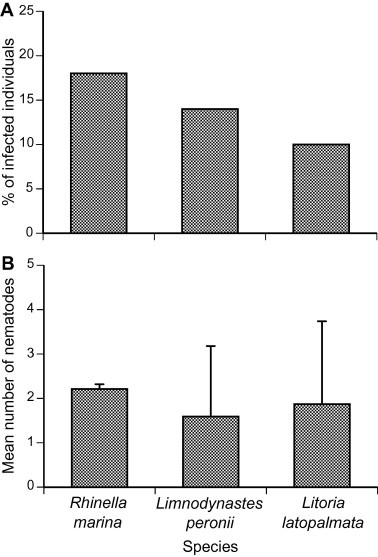
(A) Prevalence (% of anurans infected) and (B) intensity (mean number of cysts and worms per infected host) of parasitic larval nematodes in cane toads and native anurans from northern NSW. Bars represent standard errors.

**Fig. 4:**
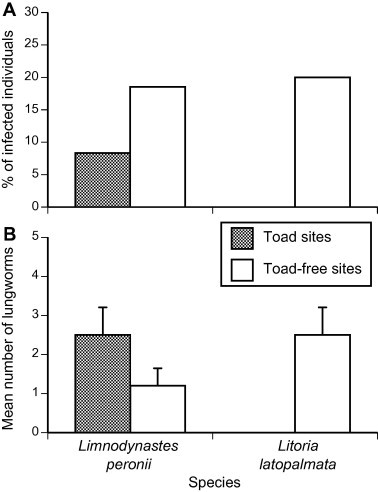
(A) Prevalence (% of anurans infected) and (B) intensity (mean number of cysts and worms per infected host) of parasitic nematodes in anurans from cane toad-present, and cane toad-absent areas in northern NSW. Bars represent standard errors.

The overall prevalence of gastric-encysting and third-stage larval nematodes in anurans from all sites did not differ significantly among species (*χ*^2^_2_ = 0.94, *p* = 0.62; Fig. 3). Cane toads were infected at higher intensities than *L. peronii* overall (one-factor ANOVA, *F*_2,14_ = 7.15, *p* = 0.007), but *L. peronii* and *L. latopalmata* were infected at similar intensities (Tukey’s HSD, *p* > 0.05). The numbers of parasitic larval nematodes detected per host showed similar patterns to *Rhabdias* lungworm infections. That is, cane toads contained more larval nematodes than did native frogs, and frogs collected from “toad-absent” sites tended to be more heavily infected than were frogs of the same species from “toad-present” sites (Fig. 4). Overall, larval nematode numbers in cane toads were similar to those in native frogs from “toad-absent” sites. In both *L. peronii* and *L. latopalmata*, larval nematode prevalence was higher in native frogs from “toad-present” areas than in conspecifics from “toad-absent” sites (logistic regression: *χ*^2^_1_ = 4.33, *p* < 0.04; see Fig. 4A), but was not significantly affected by frog species (*χ*^2^_1_ = 0.01, *p* = 0.92) or body mass (*χ*^2^_1_ = 2.66, *p* = 0.10). The intensity of infection with larval nematodes in frogs from “toad-present” sites did not differ significantly from those in “toad-absent” sites (ANOVA: mass effect *F*_1,0.56_ = 1.28, *p* = 0.55; species effect *F*_1,0.69_ = 0.40, *p* = 0.68; toad presence *F*_1,4.17_ = 0.20, *p* = 0.68), and nor did overall parasite burden due to these taxa (mass effect *F*_1,56_ = 2.51, *p* = 0.12; species effect *F*_1,56_ = 0.32, *p* = 0.57; toad presence *F*_1,56_ = 2.50, *p* = 0.12). We detected no larval nematodes in *L. latopalmata* from “toad present” sites (Table 3, Fig. 4); and in *L. peronii*, there was a twofold difference in mean intensity of infection from cane toad-present sites versus toad-absent sites. Interestingly, third stage larvae of the genus *Physaloptera* were detected in *L. peronii* both from cane toad-present sites (*n* = 1), and cane toad-absent sites (*n* = 1). These were detected under the throat (*n* = 1), under the left armpit (*n* = 1), and near the groin (*n* = 2). *Physaloptera* nematodes have not been previously reported from *L. peronii* (Barton, 1994), thus these records comprise a host extension for that parasite taxon.

### 3.3 Chytrid fungus

PCR analysis of swabs detected significant chytrid infection in one *L. peronii* (mean counts from three replicates = 60.6 zoospore genomic equivalents) from a “toad-absent” site (Tucabia: Tables 1 and 3), and lower numbers in one cane toad (mean = 2.9 zoospores; from Broom’s Head: Tables 1 and 3). Trace amounts of zoospores were also recorded from one of three replicate samples from a single *L. peronii* (mean = 0.3 zoospores; from a “toad-present’ site; Sugar Glider: Tables 1 and 3) and a single *L. latopalmata* (mean = 0.2 zoospores; from a “toad-present” site; Murray’s: Tables 1 and 3). Thus, these sample sizes are too low for statistical analysis.

### 3.4 Myxozoans

*Cystodiscus* spores were detected too rarely to allow for robust statistical analysis. Spores were only detected in one *L. peronii* and one *L. nasuta*; both from a “toad-absent” site (Swan Bay Rd: Tables 1 and 3).

## 4 Discussion

In summary, we examined 144 anurans (44 cane toads and 100 frogs of three species), and compared parasite prevalence and intensity in frogs sympatric with cane toads, to frogs from sites where cane toads were absent. Laboratory experiments and field surveys on tropical Australian anurans have concluded that cane toads are unlikely to transmit rhabditoid parasites to native frogs (Phillips et al., 2010; Pizzatto et al., 2010; Pizzatto and Shine, 2011). Likewise, our studies at the southern front of the cane toad invasion suggest little if any transmission of parasites from cane toads to sympatric native frogs; indeed, the arrival of cane toads may reduce rather than increase parasite burdens of native frogs. Our results also differ from those of the tropical studies in showing that even at the invasion front; southern cane toads carry many lungworms (*Rhabdias*). That difference is consistent with mathematical models of transmission probability that attribute the lack of lungworms in tropical invasion-front toads to the rapid rate of spread of the invader in that system (Phillips et al., 2010). The apparent lack of transfer of parasites from toads to Australian frogs likely reflects a lack of co-evolutionary history. Species of the family Bufonidae are native to every region of the world except the Antarctic, Madagascar, and the Australia-New Guinea plate (Pramuk et al., 2008). The historical absence of toads from Australia has resulted in a situation whereby native parasites favour local hosts, and introduced parasites remain host-specific (Gandon and Michalakis, 2002; Poulin, 2007; Dunn, 2009). Nonetheless, parasites were present in both cane toads and native frogs. Below, we examine patterns in parasite prevalence and intensity in more detail.

### 4.1 *Rhabdias*

*Rhabdias* lungworms were found in all of the anuran species that we examined, with cane toads containing a significantly higher intensity overall (Fig. 1; Table 3). Native frogs from toad-inhabited areas had fewer *Rhabdias* than did frogs from areas without toads, suggesting that the presence of cane toads has reduced rather than increased rates of *Rhabdias* infection in frogs (Fig. 2). In fact, the toads and frogs likely have different species of *Rhabdias* that can only be distinguished using genetic methods (Dubey and Shine, 2008). Although the *Rhabdias* species infecting wild cane toads was previously identified as *R. hylae* (Johnston and Simpson, 1942; Barton, 1998), a parasite native to Australian frogs, genetic tests indicate that cane toads are infected with *R. pseudosphaerocephala* (Dubey and Shine, 2008). Most native frogs from tropical Australia that were experimentally infected with *R. pseudosphaerocephala* larvae survived, and did not sustain the infection (Pizzatto and Shine, 2011). However, certain species (*Litoria caerulea*, *Litoria dahlii*, and *Cyclorana longipes*) were able to retain even more lungworms than did cane toads. These results suggest that, under the right conditions, native frogs may be susceptible to *R. pseudosphaerocephala* infection (Pizzatto et al., 2012). However, vulnerability differs even among closely-related frog species. In our own study, we found similar lungworm prevalence and intensity in *L. peronii* and *L. latopalmata*, and a reduced prevalence (and thus, overall parasite burden) associated with cane toad presence.

Counter to our initial predictions, these two species of native frogs not only are not at risk from invading cane toads introducing parasites; the toads may actually reduce rather than increase the parasite burdens of native frogs. The mechanisms underlying this unanticipated effect warrant further research. Plausibly, cane toads might selectively consume insects that otherwise would transmit parasites to native frogs; or cane toad parasites might trigger changes to frog immune systems that make the frog more capable of dealing with infective larvae of native parasite taxa. More likely, many native parasites are taken up by cane toads but fail to survive because of the host’s ability to mount an effective immune defence. Experimental studies show that native frogs can deal effectively with the toad’s lungworm species in this way (Pizzatto et al., 2010), suggesting that cane toads may be similarly capable of dealing with the parasites of native frogs. Keesing et al. (2006) suggested that higher host diversity actually reduces disease risk, especially when competent hosts are abundant. The invasion of cane toads into native ecosystems may reflect a similar scenario as has been documented with introduced fish (Kelly et al., 2009), whereby the invaders over- procure parasites that then fail to develop within the introduced host, consequently reducing infection rates of native fauna. Analogously, Freeland et al. (1986) suggested that cane toad invasion has imperilled a tapeworm of native frogs, by breaking its usual transmission to higher-order vertebrate predators.

The prevalence of *R. pseudosphaerocephala* was as high (70%) in cane toads at the southern invasion front as in well-established cane toad populations in Queensland (80%; Barton, 1998). Studies of the cane toad invasion in tropical Australia indicate that cane toads move almost three times as fast as they do in subtropical populations (Phillips et al., 2006; Urban et al., 2007), and these toads are parasite free for at least a year or two after initial colonisation. Our results support the prediction from Phillips et al. (2010) that parasitic lungworms will be present in the cane toad’s southern invasion front. The slow speed of the subtropical cane toad invasion in north-eastern NSW compared to the tropical cane toad invasion allows toads to establish denser populations before spreading, and thus offers parasites a greater host density at the range-edge. Kelehear et al. (2012) found that *R. pseudosphaerocephala* near the range-edge of toads had evolved significant shifts in life-history traits (such as increased body size, faster maturation and smaller quantities of larger eggs) in response to low host-densities (and thus, longer exposure times in harsh external environments prior to finding a new host). However, this may not be the case with the southern cane toad invasion. Nematodes are particularly vulnerable to desiccation (Perry, 1999), a major threat to exposed free-living lungworm larvae in tropical Australia. North-eastern NSW offers lower ambient temperatures and more consistent rainfall than do the tropics of the Northern Territory (Bureau of Meteorology, 2012), potentially enhancing the survivorship of *R. pseudosphaerocephala* in the external environment.

### 4.2 Other nematodes

Cane toads host more than 80 species of helminths across their entire geographic range, of which at least 30 are nematodes (Speare, 1990; Barton, 1994; Kelehear and Jones, 2010). Thirteen species of nematodes have been recorded in Australian populations of cane toads (Mawson, 1972; Freeland et al., 1986; Barton, 1994; Dubey and Shine, 2008; Kelehear and Jones, 2010). At least 70 species of other parasitic helminths have been described and detected in native Australian frogs (Barton, 1997, 1999), and 15 have been detected in introduced cane toads (Barton, 1997; Kelehear and Jones, 2010). We did not identify the parasites responsible for the formation of gastric cysts to species level, but we recorded cyst numbers. Kelehear and Jones (2010) study on gastric encysting nematode larvae of Northern Territory cane toads and frogs (*Cyclorana australis*, *Limnodynastes convexiusculus*, *L. caerulea*, *L. dahlii*, *L. nasuta*, *Litoria rothii* and *Litoria tornieri*) identified eight species of nematodes across all anuran taxa. All species were present in cane toads, and six of the eight were present in native frogs; however, native frogs were never detected with more than two different nematode species concurrently, whereas cane toads could bear up to three. Kelehear and Jones (2010) found that gastric cysts were more prevalent in cane toads (58% of 45 specimens) than in native frogs (23% of 66). In our own study, we found gastric cysts in toads and all three species of native frogs, but prevalence was lower in cane toads of the southern invasion front (19% of 44 specimens) than was recorded in the tropical invasion. Surprisingly, gastric nematode prevalence was similar for cane toads versus native frogs collected from toad-absent sites, whereas natives in sympatry with cane toads exhibited a lower prevalence. These results suggest that the arrival of cane toads does not increase the rate at which frogs are infected with nematodes, nor is it likely that cane toads act as reservoirs for these frog nematodes. Indeed, cane toads may reduce gastric nematode infection in native frogs, by selectively consuming the invertebrate taxa responsible for transmitting those parasites, or by diluting parasite populations from native hosts (see above).

### 4.3 Chytrid fungus

Cane toads have been hypothesised to carry *B. dendrobatidis*, and hence spread the fungus to native frogs (Berger et al., 1999; Murray et al., 2011). The only study to date on this topic was that of Phillips et al. (unpublished data), who tested 100 adult cane toads from Cairns and Normanton, Queensland; none exhibited *B. dendrobatidis* infection (see Shine, 2010). However, the warmer climates of tropical Australia are unsuitable for *B. dendrobatidis* growth and persistence, as the fungus can only survive in cooler, temperate climates (Van Sluys and Hero, 2009). In our study, *B. dendrobatidis* was only detected on a single cane toad, two *L. peronii* and one *L. latopalmata*. So, cane toads can indeed serve as a host for chytrid, but the low prevalence of infection in our study, and the presence of *B. dendrobatidis* in areas without cane toads, precludes any firm conclusions. As the native frog populations south of the cane toad invasion already contain *B. dendrobatidis* (Kriger et al., 2007), cane toads are unlikely to imperil native frogs via the transfer of chytrid.

### 4.4 Myxozoans

Surveys in eastern Australia first detected myxosporean spores within the gall bladders of Australian cane toads in 1983, and native frogs (*Limnodynastes* sp., *Litoria* sp., *Mixophyes fasciolatus*, and *Uperolia laevigata*) in 1985 (Delvinquier, 1986). *M. immersum* is a myxosporean parasite from the gall bladders of South American amphibians, and was unknown in Australia prior to the cane toad’s introduction to Australia in 1935; nor was it present in native frogs outside of the cane toad’s invasion front (Delvinquier, 1986). Hartigan et al. (2010) discussed the possibility that *M. immersum* was introduced to Australia with cane toads, based on an examination of the gall bladders of 112 museum-preserved frog specimens. Spores were only detected in frogs collected post-1966, supporting the ‘cane toad introduction theory’. However, further examination suggested that Hawaiian cane toads (the source population of Australian cane toads) were *Myxidium*-free, and that the parasites of Brazilian cane toads are genetically distinct from the Australian species (Hartigan et al., 2011). This falsification of the original ‘cane toad introduction theory’ suggested instead a ‘cane toad reservoir and spill-back’ theory: that is, *Myxidium* was present but in low prevalence before cane toad introduction (hence no detection prior to 1966); cane toads then acquired *Myxidium* from native frogs and assisted its spread. In more recent work, Hartigan et al. (2012a) have revealed another complexity: there are more species of *Myxidium* in native Australian frogs than was first realised; two new species of myxozoa (*C. axonis* and *C. australis*, previously assumed to be *M. immersum*) have now been described (Hartigan et al., 2012a).

Hartigan et al. (2011) detected a high prevalence of myxospores (42% of 82) in the gall bladders of cane toads from northern NSW, whereas we did not detect any. We found myxospores only within the gall bladder of a single *L. peronii* and *L. nasuta*, both species known to carry this parasite (Freeland, 1986). However, myxozoans are not restricted to amphibian gall bladders; they may occur also within the brain, liver and testes (Hartigan et al., 2012a,b). Our microscopic analysis of gall bladder contents may not have detected all cases of occurrence. Nonetheless, the lack of any trend in myxozoan numbers in native frogs as a function of toad presence casts doubt on the idea that toads increase *Cystodiscus* infection rates in native frogs.

### 4.5 Conclusions

Cane toads are continuing to spread down the east coast of Australia (Urban et al., 2007) and will threaten populations of large native predators, as they have in tropical Australia (Shine, 2010). However, our data are more reassuring in terms of the potential impact of invading toads via their role in spreading novel parasites to native frogs. In summary, we found no evidence to suggest that cane toads either transfer novel parasites to native frog populations, or act as a reservoir for native parasites to ‘spill-back’ into native frogs. The apparent lack of transfer of parasites from frogs to cane toads also means that we cannot expect native parasites to slow the cane toad advance. Importantly, host-parasite relationships can shift through time through rapid co-evolution, or through shifts in environmental factors. Thus, future work could usefully reassess parasite prevalence and intensity in this system as a function of time since toad invasion, or as a function of shifts in climatic and landscape factors. Importantly, our surveys provide baseline data that will facilitate the interpretation of future studies on this topic.

## Figures and Tables

**Table 1:** Sites, co-ordinates, a brief description and number of anurans collected from the northern coast of NSW, Australia, between February and October 2012.

Site name	Co-ordinates	Months	*Rhinella marina*	*Limnodynastes peronii*	*Litoria latopalmata*	*Litoria nasuta*	Brief site description
Brooms Head^a^	56 530874 E, 6728518 N	April	9	–	–	–	Coastal heath and woodland, ephemeral water only
Sugar Glider^a^	56 554446 E, 6861349 N	May	4	10	–	1	Urban and Melaleuca swamp
Harry’s/Lewis Lane^a^	56 522998 E, 6752264 N	May	8	2	2	–	Open Eucalypt forest
Murray’s^a^	56 548851 E, 6866221 N	May	2	–	8	–	Open Eucalypt forest
SASS^a^	56 555104 E, 6860927 N	February	4	2	–	–	Urban and Melaleuca swamp
Cudgera^a^	56 555291 E, 6861161 N	May	12	–	–	–	Urban and Melaleuca swamp
Byrill Creek Rd^a^	56 521083 E, 6854567 N	April	4	–	1	–	Urban and low closed forest
Clothiers Creek Rd^a^	56 548649 E, 6866258 N	May	1	1	–	–	Urban, open eucalypt woodland and Melaleuca swamps
Numulgi Rd^a^	56 53111 E, 6817863 N	April	–	9	–	–	Semi-rural pastureland
Road to Brooms Head	56 523838 E, 6736106 N	May, October	–	12	–	17	Semi-rural and Eucalypt woodland
Swan Bay Rd	56 527013 E, 6777881 N	October	–	7	–	1	Semi-rural and woodland
South of Casino	56 500633 E, 6791150 N	April	–	8	–		Semi-rural and Eucalypt woodland
Tucabia	56 510311 E, 6711609 N	August	–	1	–	–	Semi-rural and Eucalypt woodland
Minyumai Rd	56 529888 E, 6772027 N	October	–	–	10	8	Semi-rural and Eucalypt woodland

a Indicates cane toad presence.

**Table 2:** Species, sex, body sizes, and brief description of anurans collected and examined for parasites, from the northern coast of NSW, Australia, between February and October 2012.

Species	Sex	Cane toads present			Cane toads absent			Brief species description
		*n*	Mean SUL (mm)	Mean mass (g)	*n*	Mean SUL (mm)	Mean mass (g)	
*Rhinella marina*	M F	26 18	85.33 74.93	101.87 69.26	– –	– –	– –	Ground dwelling, prefers still waterbodies
*Limnodynastes peronii*	M F Unk	4 19 1	42.18 45.67 48.7	6.83 9.09 11.86	11 17 –	36.25 41.15 –	4.73 6.59 –	Ground dwelling, still and moving waterbodies
*Litoria latopalmata*	M F Unk	4 7 –	34.43 35.31 –	4.16 4.26 –	7 2 1	30.65 34.92 30.47	2 4.5 3	Ground dwelling, still and moving waterbodies
*Litoria nasuta*	M F Unk	– 1 –	– 32.3 –	– 4.22 –	12 12 2	37.32 38.14 28.35	4.67 4.98 1.17	Ground dwelling, slow moving and still waterbodies

SUL = snout-urostyle length; F = female; M = male; Unk = unknown (gonads destroyed).

**Table 3:** Species, numbers, body sizes, and parasite prevalence and intensity of anurans collected from the northern coast of NSW, Australia, between February and October 2012.

	*Rhinella marina* (*n* = 44)	*Limnodynastes peronii*^a^ (*n* = 24)	*Limnodynastes peronii* (*n* = 28)	*Litoria latopalmata*^a^ (*n* = 11)	*Litoria latopalmata* (*n* = 10)	*Litoria nasuta*^a^ (*n* = 1)	*Litoria nasuta* (*n* = 26)
Mean size (SUL)	81.08	45.22	39.22	34.99	31.90	32.3	37.01
Mean mass (g)	88.53	8.83	5.86	4.22	2.6	2.58	4.54
*Rhabdias* prevalence (%)	70.45	36.36	38.46	18.18	50	0	16
Mean # *Rhabdias*	16.13	1.38	3	1	1.2	0	1.5
Max # *Rhabdias*	63	3	5	1	2	0	3
Min # *Rhabdias*	1	1	1	1	1	0	1
Nematode prevalence (%)	19.05	8.33	18.52	0	20	0	20.83
Mean # nematode	4	2.5	1.2	0	2.5	0	3
Max # nematode	7	3	2	0	3	0	4
Min # nematode	2	2	1	0	2	0	1
Chytrid prevalence (%)	2.27	4.17	3.57	9.09	0	–	–
Mean # zoospores	2.94	0.3	60.59	0.23	0	–	–
Max # zoospores	6.05	0.9	73.42	0.7	0	–	–
*Myxidium* prevalence (%)	0	0	3.57	0	0	0	5

“Nematode” = gastric-encysting, larval nematodes, species unknown; SUL = snout-urostyle length.

a Indicates cane toad presence.
